# Polymeric Micelles for the Enhanced Deposition of Hydrophobic Drugs into Ocular Tissues, without Plasma Exposure

**DOI:** 10.3390/pharmaceutics13050744

**Published:** 2021-05-18

**Authors:** Ijeoma F. Uchegbu, Jan Breznikar, Alessandra Zaffalon, Uche Odunze, Andreas G. Schätzlein

**Affiliations:** 1 UCL School of Pharmacy, London’s Global University, 29–39 Brunswick Square, London WC1N 1AX, UK; breznikar7@gmail.com (J.B.); alessandra_zaffalon@hotmail.it (A.Z.); uchechukwu.odunze.14@alumni.ucl.ac.uk (U.O.); a.schatzlein@ucl.ac.uk (A.G.S.); 2 Nanomerics Ltd. New Bridge Street House, 6th Floor, 2 London Wall Place, London EC2Y 5AU, UK

**Keywords:** cyclosporine A, Molecular Envelope Technology (MET), N-palmitoyl-N-monomethyl-N,N-dimethyl-N,N,N-trimethyl-6-O-glycolchitosan (GCPQ), eye, penetration enhancer

## Abstract

Commercial topical ocular formulations for hydrophobic actives rely on the use of suspensions or oil in water emulsions and neither of these formulation modalities adequately promote drug penetration into ocular tissues. Using the ocular relevant hydrophobic drug, cyclosporine A (CsA), a non-irritant ocular penetration enhancer is showcased, which may be used for the formulation of hydrophobic actives. The activity of this penetration enhancer is demonstrated in a healthy rabbit model. The Molecular Envelope Technology (MET) polymer (N-palmitoyl-N-monomethyl-N,N-dimethyl-N,N,N-trimethyl-6-O-glycolchitosan), a self-assembling, micelle-forming polymer, was used to formulate CsA into sterile filtered nanoparticulate eye drop formulations and the stability of the formulation tested. Healthy rabbits were dosed with a single dose of a MET–CsA (NM133) 0.05% formulation and ocular tissues analyzed. Optically clear NM133 formulations were prepared containing between 0.01–0.1% *w*/*v* CsA and 0.375–0.75% *w*/*v* MET polymer. NM133 0.01%, NM133 0.02% and NM133 0.05% were stable for 28 days when stored at refrigeration temperature (5–6 °C) and room temperature (16–23 °C), but there was evidence of evaporation of the formulation at 40 °C. There was no change in drug content when NM133 0.05% was stored for 387 days at 4 °C. On topical dosing to rabbits, corneal, conjunctival and scleral AUC_0–24_ levels were 25,780 ng.h g^−1^, 12,046 ng.h g^−1^ and 5879 ng.h g^−1^, respectively, with NM133 0.05%. Meanwhile, a similar dose of Restasis 0.05% yielded lower values of 4726 ng.h/g, 4813 ng.h/g and 1729 ng.h/g for the drug corneal, conjunctival and scleral levels, respectively. NM133 thus delivered up to five times more CsA to the ocular surface tissues when compared to Restasis. The MET polymer was non-irritant up to a concentration of 4% *w*/*v*. The MET polymer is a non-irritant ocular penetration enhancer that may be used to deliver hydrophobic drugs in optically clear topical ocular formulations.

## 1. Introduction

The topical ocular delivery of drugs is usually accomplished using eye drop formulations; however, these formulations have a short ocular residence time, draining through the nasolacrimal duct within 1–3 min [1]. Although ocular clearance may be delayed by up to 30–50 min through the inclusion of viscosifying polymer excipients [1], eye drops are largely inefficient at delivering drugs to the tissue due to the small volume of the eye drop (<50 μL), the rapid clearance of the formulation from the ocular surface and the water and lipid barriers that make up the tear film [2]. Strategies to improve permeation of molecules into the ocular tissues are thus highly desirable. Various nanosystems have been described as experimental formulation agents [3]. However, usually for clinical applications, the formulation of hydrophobic drugs into topical ocular formulations is either accomplished by using suspensions or emulsions, both of which are opaque and transiently interfere with vision. There is thus a requirement for transparent ocular formulations for hydrophobic drugs [4], that enhance drug transport into the ocular tissues and, importantly, are non-irritant. Such a formulation is presented using the hydrophobic cyclosporine A (CsA, water solubility = 4–134 µg/mL, with a lower solubility at 37 °C–4 µg/mL and a higher solubility at 10 °C–134 µg/mL) [5] as the model drug. 

CsA is an immunosuppressant that inhibits interleukin 2 activation of lymphocytes and thus reduces their attack on the exocrine epithelia of the lachrymal glands; this results in increased tear secretion and increased tear film stability [6]. CsA also decreases apoptosis and, as such, increases the density of mucus producing goblet cells, leading to a restoration of the damaged ocular epithelium [6]. In patients diagnosed with Dry Eye Syndrome, CsA is effective at increasing ocular surface goblet cell density and tear fluid volume from the accessory lachrymal glands [7]. The efficacy of topical CsA in controlling symptoms has been confirmed in Dry eye Disease [7] and Sjörgren’s Syndrome [8]. The ability of topical ocular CsA to improve the clinical signs of Dry Eye disease has also been shown [9]. CsA is an extremely hydrophobic drug that is formulated in an oil-in-water emulsion in the approved products Restasis, marketed by Allergan [10] and Ikervis [11], and marketed by Santen.

With these commercial formulations there is the need for frequent dosing, e.g., with Restasis, which is dosed as one drop (28 μL, 14 μg) at least twice daily [12] (a total daily dose of at least 28 μg per day). There is also the risk of patients (19–27%) experiencing eye pain with Ikervis [9] and Restasis [13,14,15]. Restasis must be administered at least twice a day to achieve therapeutic concentrations in the target ocular tissues and the product is also associated with burning and stinging [13,14,15]. There are even strategies, such as the use of corticosteroids, proposed to limit the burning and stinging [15]. With the Ikervis vehicle, an oil in water emulsion, 5.9% of patients abandoned the treatment with the vehicle in blinded clinical trials due to ocular treatment emergent adverse events [9] and with the Restasis vehicle, 11.8% of patients reported eye pain, burning or stinging [16]. Despite the clinical benefit achieved with cyclosporine A emulsions, the ocular discomfort is the most common reason for patients discontinuing the therapy [15]. As many as 27% of patients on Restasis complain of burning and stinging with the therapeutic [7]. The European Medicines Agency’s approval of Ikervis for the treatment of severe keratitis in Dry Eye patients, that does not respond to artificial tears, requires Santen to institute and maintain a pharmacovigilance programme as 19% of patients suffer from eye pain and 18% from eye irritation along with other ocular adverse effects [11]. Formulations made using a non-irritant vehicle (see below), such as the Molecular Envelope Technology (MET–N-palmitoyl-N-monomethyl-N,N-dimethyl-N,N,N-trimethyl-6-O-glycolchitosan) polymer (GCPQ) [4], would thus be beneficial. 

However, more importantly, and constituting the main focus of this study, is that some patients do not benefit from Restasis when administered twice daily, and such patients benefit from more frequent dosing of Restasis (to increase the level of drug in the tissues) [12]. Dosing Restasis at one drop (28 μL) 3–4 times a day, as opposed to twice a day, produced clinical benefit in 68% of a cohort of 21 patients who were previously refractory to twice daily doses of Restasis, although 13.6% of these patients complained of stinging and irritation with this regimen [12]. Patients that responded to this intensive dosing regimen received between 42 and 56 μg of CsA per day. It appears that increasing drug absorption in a well-tolerated formulation would be of benefit to these erstwhile refractory patients. Additionally, increased drug absorption would benefit ocular CsA patients responding to the twice daily regimen as these latter patients would be able to reduce dosing frequency from twice a day to once a day and, thus, reduce the number of ocular pain events. 

Increasing drug absorption is the main purpose of this study. The MET polymer delivers prednisolone more efficiently to the aqueous humour when compared to a commercial formulation of the drug [4]. The MET polymer is a mucoadhesive [17] self-assembling nanoparticle (micelle) forming polymer, with pendant hydrophobic groups [18] and a CMC in the low micromolar region [17,19]. It has been previously shown that amphiphiles with pendant hydrophobic groups are able to encapsulate CsA within aqueous disperse phases at both physiological (where CsA has very poor solubility –4 µg/mL) and refrigeration (where CsA has a solubility of 134 µg/mL) temperatures [20]. The MET polymer is non-irritant in a rabbit model at low concentrations (see below) and a proven penetration enhancer: it produces similar aqueous humour levels when applied at 1/10th of the dose of the commercial prednisolone formulation [4] and up to 18 times more tacrolimus in ocular tissues when compared to drug levels obtained with a commercial tacrolimus formulation [21]. The MET polymer forms positively charged nanoparticles which are mucoadhesive [17] and it is hypothesized that this mucoadhesion contributes to the penetration enhancement. The MET polymer is a chitosan amphiphile and others have reported on the penetration enhancement of ocular chitosan formulations by way of tight junction opening [22]; however, the MET polymer does not open tight junctions in the concentrations employed [17].

In summary, within this work, the MET polymer was tested as a formulation excipient to produce CsA eye drops in an aqueous, oil-free formulation, with the aim of improving the penetration of CsA into the surface ocular tissues. Higher ocular tissue levels could increase the response rates of patients that are refractory to the normal twice daily dose of Restasis [12] as well as reduce dosing frequency to once a day, with 0.05% *w*/*v* CsA, in other patients responsive to the twice daily Restasis dose, and in effect limit the incidence of ocular pain.

## 2. Materials and Methods

### 2.1. Materials

All chemicals and reagents were obtained from Sigma Aldrich (Poole, UK) unless otherwise stated. Organic solvents were obtained from Fisher Scientific (Loughborough, UK).

### 2.2. Formulation Preparation

A number of formulations were prepared in order to select the best formulation for the preclinical rabbit study. The various cyclosporine formulations prepared (NM133) are shown in Table 1.

#### 2.2.1. Method I—High Pressure Homogenization (Non-Sterile with Glycerol)

The MET polymer (Lot Number: GCPQLC2Sep13Deprot, 0.75% *w*/*v*, obtained from Nanomerics Ltd., London, UK) was dispersed in aqueous glycerol (3.1% *w*/*v*). The polymer was allowed to disperse by gently shaking on an orbital shaker for at least 2 h. Once the polymer was completely dispersed, the dispersion was filtered using a 3.1 µm syringe filter. The polymer dispersion above was add to CSA powder (the CSA was 2× times the target amount (0.1% *w*/*v*) to account for any losses during processing) with vortexing and the mixture was subsequently processed for 30 cycles at 18,000 psi using a high pressure homogenizer (Avestin C5 Emulsiflex, Biopharma Group, Winchester, UK). After preparation the pH was adjusted to pH = 7.4 using NaOH (1 M). After this step, the formulation was stored for at least 24 h at 5 °C. It was then analyzed by HPLC (see below) and finally diluted with a MET polymer dispersion (0.75% *w*/*v*) in glycerol (3.1% *w*/*v*). This MET polymer dispersion had been previously adjusted to pH = 7.4 and filtered using 3.1 µm syringe filters. This MET polymer dispersion was used to make up the formulation to the right strength.

#### 2.2.2. Method II—High Pressure Homogenization (Sterile with Glycerol)

Formulations prepared for the 28-day stability study were prepared as outlined below. The MET polymer (Lot Number: GCXP32Q17JB) was dispersed (0.75% *w*/*v*) in a solution of glycerol in water (2.7% *w*/*v*) with shaking for at least 2 h, until completely dispersed. The polymer solution was added to CsA powder (twice as much as the target concentration). CsA was dispersed by initially vortexing the mixture followed by stirring with a magnetic stirrer at 4 °C for 3–5 h and the mixture was subsequently processed for 15 cycles at 19,000 psi using a high pressure homogenizer. The pH was adjusted to pH = 7.0 using NaOH (1 M) and the formulation stored in the fridge for 24 h to stabilize the concentration of CsA encapsulated. The formulation was then analyzed by HPLC (please see below) and made up to full strength using the MET polymer (0.75% *w*/*v*) in glycerol (2.7% *w*/*v*) adjusted to pH = 7.0 with NaOH (1 M). The formulations were then sterile filtered (0.22 µM) into 3 mL Steri-Dropper^TM^ polyethylene sterile eye-dropper bottles and assessed for sterility using the British Pharmacopoeia method [23].

#### 2.2.3. Method III—High Pressure Homogenization (Non-Sterile-Phosphate Buffered Saline)

For formulations prepared in phosphate buffered saline (PBS, pH = 7.4), Method II was used, with the exception that high pressure homogenization was carried out at 20,000 psi for 30 cycles and the pH was not adjusted using NaOH (1 M).

#### 2.2.4. Method IV—Probe Sonication (Non-Sterile with Glycerol)

Probe sonication was also used to prepare formulations in order to evaluate this laboratory method. The MET polymer (15 mg, Lot Number-GCP19Q12 for NM133 0.08%A, GCP37Q23 for NM133 0.08%B and GCP19Q9 for NM133 0.1%) was dispersed in glycerol (1% *v*/*v*, 2 mL) and to this was added CsA powder (2 mg); the mixture was then probe sonicated using an MSE Soniprep 150 sonicator (MSE UK Ltd.) at an amplitude of 8 microns for 20 min in an ice bath. After sonication, the mixture was placed in the fridge at 4 °C overnight. The following day, the mixture was filtered using a 0.22 μm filter and reserved for HPLC analysis (please see below). These formulations were stored in the fridge and drug content was monitored over 387 days.

### 2.3. Formulation Characterisation

#### 2.3.1. Drug Content

Drug content was analyzed using a high performance liquid chromatography (HPLC) assay. For the HPLC assay, aliquots (100 μL) were diluted with an equivalent volume of methanol, the solution was filtered (0.22 μm) and the filtrate injected onto a C18 reverse phase Onyx monolithic column (100 × 4.6 mm). A gradient mobile phase was used as follows: 0 min—acetonitrile, water = 60:40 *v*/*v*; 4 min—acetonitrile, water = 5:95 *v*/*v*; 7.5 min—acetonitrile, water = 60:40 *v*/*v*. The run time was 10.5 min, the retention time for CsA was 4.9 min, the flow rate 1.5 mL min^−1^, the injection volume was 20 μL and the column temperature was 40 °C. Analytes were monitored at a UV absorption wavelength of 210 nm (Figure S1 in Supplementary Information). A standard curve was prepared using cyclosporine A concentrations ranging from 10 to 1250 μg mL^−1^ in methanol (y = 26527x + 370.25 r^2^ = 0.9975, limit of quantification = 0.01 mg mL^−1^). The HPLC system was an Agilent 1200 Series (Agilent Technologies Ltd., Stockport, UK) and the data were analyzed by Agilent ChemStation software, version 07/09 (Agilent Technologies Ltd., Stockport, UK).

#### 2.3.2. MET Content

The MET polymer was analyzed at the end of the stability studies. Samples were chromatographed over a Waters Acquity BEH Amide, 50 × 2.1 mm, 1.7 µm UHPLC column using the following gradient for isopropyl alcohol (A) and 0.2% *v*/*v* formic acid in water, acetonitrile (70:30) (B): 0 min—A, B = 90:10 *v*/*v*; 7 min—A, B = 0:100 *v*/*v*; 14 min—A,B = 90:10 *v*/*v*. The run time was 15 min and the MET retention time was 11.2 min. Chromatography was driven by an Agilent Technologies 1600 series HPLC system at a flow rate 0.3 mL min^−1^. The column temperature was set at 35 °C and the MET polymer was detected using an Agilent evaporative light scattering detector (ELSD) using a nebulizer temperature of 70 °C, evaporator temperature of 60 °C and a gas flow (N_2_) rate of 1.6 L min^−1^. Samples for the calibration curve (y = 1828.5x − 436.09 r^2^ = 0.9936, limit of quantification = 0.5 mg mL^−1^) were prepared in methanol at concentrations of 0.25, 0.50, 0.75, 1.0 and 1.5 mg mL^−1^. Formulation samples were diluted ten times with methanol prior to chromatography. 

#### 2.3.3. Osmolarity

The osmolarity of the formulation (100 μL) was determined using Roebling Milliosmol Osmometer (Geminibv, Apeldoorn, The Netherlands) coupled with a digital display and a freezing needle. The machine was calibrated before each measurement with 300 mOsm/Kg reference standards solution (Reagecon Diagnostics Ltd., Clare, Ireland). The measurements were conducted in triplicate.

#### 2.3.4. Electron Microscopy

The formulation was visually observed and examined by transmission electron microscopy (TEM) [17] for the presence of drug crystals. TEM was carried out using a Philips/FEI CM120 Bio Twin electron microscope (Philips, Eindhoven, The Netherlands). A drop of the formulation was dried on a copper TEM grid (300 mesh, Formvar/carbon coated) and stained with a drop of uranyl acetate (1% *w*/*v*, for negative staining). Once dried, the samples were imaged using TEM, and the representative images photographed and documented.

#### 2.3.5. Particle Size

The particle size distribution and particle zeta potential of the formulation were determined by dynamic light scattering (DLS) on a Malvern ZetaSizer Nano ZS (Malvern Panalytical, Malvern, UK). The size distribution analysis was performed at a backscattering angle of 173° and a temperature of 25 °C. An aliquot of the sample (100 μL) was placed in a disposable plastic cuvette and was subsequently loaded into the instrument without any dilution. The particle size was reported as intensity distribution, which describes the relationship between light scattering intensity and the particle hydrodynamic diameter. The mean size of the individual peaks and their corresponding percentages were determined and recorded as mean ± standard deviation (s.d.) from three independent measurements.

#### 2.3.6. Zeta Potential

The zeta potential is the electrokinetic potential in a colloidal system, and measures the effective surface particle charge in a given medium [24]. This parameter was obtained via the electrophoretic light scattering technique. An aliquot of the sample (500 μL) was loaded into the disposable folded capillary cell. Measurements were performed in triplicate, and the results were presented as mean ± s.d.

### 2.4. Formulation Stability

Storage stability of the formulations (NM133 0.05%B, NM133 0.02%A, NM133 0.01%B) was analyzed by storing the formulations for 28 days at refrigeration temperature (5–6 °C), room temperature (18–25 °C) and at 40 °C, and the formulations were analyzed for: drug content using HPLC analysis as described above, osmolarity, viscosity, particle size and zeta potential. The initial pH and visual appearance of the formulations were also recorded. 

NM133 0.08%C *w*/*v* was also subjected to freeze thaw cycles: −20 °C for 2 days, 5 °C for 2 days, 40 °C for 2 days, with the whole process repeated for a total of 3 cycles. Drug content and particle size were periodically monitored using the methods described above.

Data were analyzed by comparing all data points using ANOVA with statistical significance set at *p* < 0.05.

### 2.5. Ocular Pharmacokinetics

#### Animal Ocular Biodistribution Studies

NM133 0.05%A and NM133 0.01%A were used for the study. All animal studies were carried out under a UK Home Office license (PPL 70/8224) and approved by the local ethics committee. New Zealand albino male rabbits weighing between 2.5 and 3 kg (Harlan laboratories, Bicester, UK) were acclimatized for not less than 5 days before the experiments. The rabbits had free access to water throughout the study. The formulations (*n* = 3 animals per group) were administered to both eyes. To administer the formulations, the lower eyelid was gently pulled away from the eye globe and, using a calibrated micropipette, 25 µL of the formulations was applied in the lower conjunctival cul-de-sac. After dosing, the upper and lower eyelids were hand-held together for approximately 5 s to permit the formulations to come in contact with the cornea. Subsequently, the number of blinks in the following 60 s was recorded. At prearranged time points (0.5, 2, 4, 8, 24 h), a sample of arterial blood was taken from the marginal ear artery. Subsequently, the rabbits were culled with an IV over-dose injection of pentobarbital. The various tissues were dissected, rinsed with 0.9% NaCl solution, dried on a filter paper and stored for subsequent analysis. The eye tissues were harvested in the following order to minimize contamination: (1) aqueous humour, (2) conjunctiva, (3) vitreous humour, (4) lens, (5) cornea and (6) sclera. The tissues from both eyes were stored in the same container. Initially (2–5 h after dissection) the samples were stored in ice and then they were stored at −80 °C until analysis could be done on them.

### 2.6. Sample Analysis

The concentrations of CSA in tissues were determined using liquid chromatography-mass spectrometry (LC-MS/MS).

#### 2.6.1. Preparation of Standards

CsA stock solutions (1 mg mL^−1^) were prepared in glass vials in methanol. Working standards were prepared by serially diluting a CsA stock solution into methanol to obtain the working standards (WS) as shown in Table S1 (Supplementary Information) within a concentration range of 50 to 1,000,000 ng mL^−1^. CsA-d12 (Recipe, Munich, Germany) was used as the internal standard (IS). Stock solutions of the IS (6.25 μg mL^−1^) were prepared in acetonitrile. The IS standard solution (IS-PPT) was freshly prepared by diluting the IS stock solution with methanol to yield an IS with a concentration of 5 ng mL^−1^.

To spike blank tissues, the blank tissues were defrosted (and cut into small pieces using scissors if a solid tissue) weighed (99.0 ± 1.0 mg—or 99 μL for liquid tissue) and placed in 1.5 mL polypropylene micro-centrifuge tubes. To each tube was added a volume of the working standards WS0–WS14 (Table S2—Supplementary Information). Spiked samples were then vortexed for 10 min.

Spiked samples were then extracted by adding of IS-PPT (400 µL) and vortexing for 4 h at room temperature. Subsequently the samples were removed from the vortex and left to stand at 5 °C for 30 min before being centrifuged (5000× *g* × 10 min, MSE Micro Centaur, London, UK) The supernatant was transferred to HPLC vials and analyzed by liquid chromatography-mass spectrometry (LC-MS).

#### 2.6.2. Tissue Processing

Tissues from the ocular pharmacokinetics study were defrosted (and cut into small pieces using scissors if a solid tissue) weighed (100.0 ± 1.0 mg—or 100 μL for liquid tissue) and placed in 1.5 mL polypropylene micro-centrifuge tubes. Samples were then extracted by adding of IS-PPT (400 µL) and vortexed for 4 h at room temperature. Subsequently the samples were removed from the vortex and left to stand at 5 °C for 30 min before being centrifuged (5000× *g* × 10 min). The supernatant was transferred to HPLC vials and analyzed by LC-MS. 

#### 2.6.3. LC-MS/MS Instrumentation

Samples were analyzed over an Agilent 6400 Series Triple Quadrupole LC/MS system (Agilent technologies, Berkshire, UK) comprising a degasser (HiP Degasser 1260/G4225A), a binary pump (HiP 1260 binary pump/G1312B), an autosampler (HiP sampler 1260/G1367E), a column oven (G1316A) and a triple-quadrupole mass spectrometer (G6460A). Agilent MassHunter Workstation Software was used for system control, data acquisition and data processing.

#### 2.6.4. Chromatography Conditions

Samples (injection volume = 5 μL) were chromatographed over an Agilent Zorbax Extend-C18 50 × 2.1 mm column, pore size = 3.5 μm, equipped with a Cartridge Gemini C18 4 × 2.0 mm guard column and at a column temperature of 60 °C and a mobile phase flow rate of 600 μL min^−1^. Samples were chromatographed using the following gradient for 0.02% *w*/*v* acetic acid in water (A) and 0.02%w/v acetic acid in methanol (B): 0 min—A, B = 30:70 *v*/*v*; 1.8 min—A, B = 0:100 *v*/*v*; 2.1 min—A, B = 30:70 *v*/*v*. The run time was 3 min. The retention times for the two analytes, obtained under the above chromatographic conditions, were 2.14 min and 2.14 min for CsA and CsA-d12, respectively. 

#### 2.6.5. Mass Spectrometer Conditions

The ion source was an Agilent Jet Stream (AJS) with nitrogen as source, the scan mode was multiple reaction monitoring (MRM), the polarity was in positive ion mode, the nebulizer pressure was at 30 psi, the gas flow was set at 5 L min^−1^, the gas temperature at 340 °C, the capillary voltage was set at 5000 V, the sheath gas heater was set at 400 °C, the sheath gas flow was set at 11 L min^−1^ and the VCharging was set at 1500 V. Table S3 (Supplementary Information) presents the mass spectrometer conditions for the quantification of the analyte.

#### 2.6.6. Quantification

Samples were quantified using the calibration curves for CsA and CsA-d12, prepared as described in Table S2 (Supplementary Information).

#### 2.6.7. Pharmacokinetic Analysis

Microsoft Excel Professional Plus 2010 was used to calculate pharmacokinetic parameters. IBM SPSS Statistics was used for statistical analyses. Values below the limit of quantification (BLQ) were considered to be 0 for the calculations.

### 2.7. Statistical Analysis for Pharmacokinetics Studies

IBM SPSS Statistics was used for statistical analyses. Values below the limit of quantification (BLQ) were considered to be 0 for the calculation. At first, a 2-way ANOVA test followed by a post-test (Tukey’s HSD) were performed to test the difference between the 3 formulations throughout the entire set of time points. When a statistically significant difference existed among the three formulations, statistically significant differences within each time point were evaluated with one-way ANOVA followed by a post-test (either Tukey’s HSD or Games-Howell with equal or unequal variance, respectively). 

### 2.8. Ocular Tolerability of the MET Polymer

Groups of female New Zealand White rabbits (*n* = 3) were dosed in the left eye twice daily over 6 days with one drop of the MET polymer (0.1% *w*/*v*, 0.5% *w*/*v*, 1% *w*/*v*, 2% *w*/*v* or 4% *w*/*v*) dispersed in phosphate buffered saline (pH = 7.4), and all animals were scored for ocular irritation: 0 = no discomfort (no response), 1 = minimum discomfort (a few blinks only), 2 = mild discomfort (closing the eye after blinking), and 3 = moderate discomfort (rabbit holds eye shut).

On administration of NM133 to rabbits, the blink rate was recorded for the following 60 s immediately after administration.

## 3. Results

### 3.1. Formulation and Stability Studies

**Figure 1:**
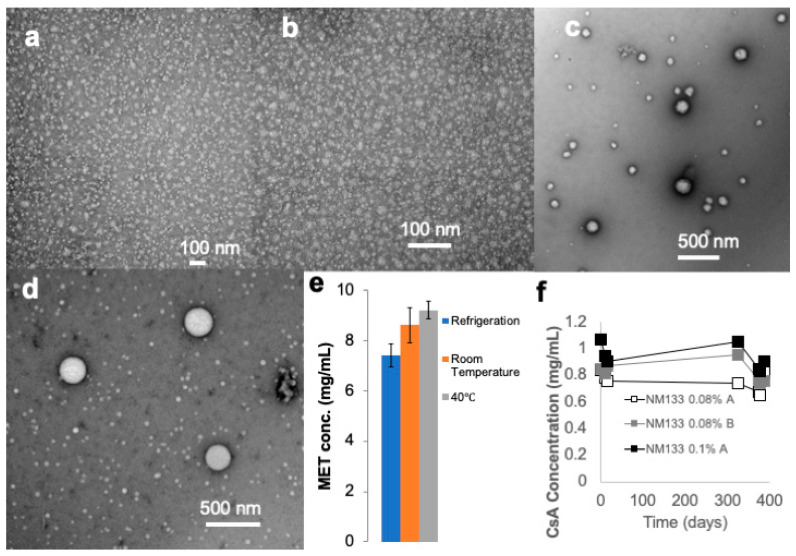
(**a**) Transmission electron micrograph (TEM) of freshly prepared NM133 0.05%C eye drops, (**b**) TEM of NM133 0.05%C after storage for 7 days at room temperature, (**c**) TEM of NM133 0.05%C after storage for 21 days at refrigeration temperature showing the polydisperse nature of NM133 formulations prepared in PBS (larger particles were seen at other time points in all storage conditions), (**d**) TEM of freshly prepared NM133 0.1%B, (**e**) MET levels in the NM133 0.02%A formulations after storage for 30 days, (**f**) Drug levels after storage (mean ± s.d. *n* = 3) of NM133 0.08%A, NM133 0.08%B and NM133 0.1%A prepared by probe sonication and after storage for 387 days at 4 °C (error bars are obscured by the data point).

MET–CsA (NM133) formulations were prepared at various strengths and characterized (Table 1). Optically clear liquid CsA eye drops were formulated using the MET polymer [4,21], in which CsA was encapsulated within MET polymer micelles (Table 2, Figure 1a,b). These formulations were prepared in two disperse phases: phosphate buffered saline (PBS, pH = 7.4) and glycerol (e.g., 2.7% *w*/*v*), and characterized (Table 2). The formulations are stabilized by the charge repulsion occurring due to the positive zeta potential on the particle surface. In the presence of sodium chloride there is charge screening, which results in a larger particle size due to particle aggregation (Table 2, Figure 1c,d). Formulations prepared in PBS were thus polydisperse (Table 2, Figure 1c,d).

### 3.2. Stability Studies

Formulations prepared in glycerol were stable for up to 28 days when stored in the fridge or at room temperature, with any significant differences in parameters such as osmolarity and viscosity not exceeding 10% of the initial value recorded at the start of the storage experiment (Table 3 and Table 4). Additionally, drug content remained stable in sample formulations for up to 387 days (Figure 1f). On storage at 40 °C, NM133 in glycerol is less stable after 28 days storage, as there are changes in drug concentration, osmolarity and zeta potential (Table 5). The formulations were stored in screw capped bottles and these changes are presumed to be due to evaporation of the formulation.

CsA has an unusual temperature solubility profile, in that it is 20 times more soluble at refrigeration temperature, when compared to its solubility at 35 °C [5]. It is thus interesting to see that the PBS formulation was stable when subjected to freezing at 2–6 °C, thawing at room temperature at 16–23 °C and heating (40 °C) cycling when held for 2 days at each temperature. The cycling was run over three six-day each cycles and the formulation was able to accommodate the reduced solubility of CsA at elevated temperature (Table 6). The glycerol formulation (NM133 0.05% A) was suitably sterilized by sterile filtration (0.22 µm) without any loss of drug content (Table 2 and Table 7); this is evidence of the sub-200 nm particle size of this formulation. After considering the particle size distribution data, the glycerol formulation prepared using Method 1 was selected for preclinical assessment in the rabbit model.

**Figure 2:**
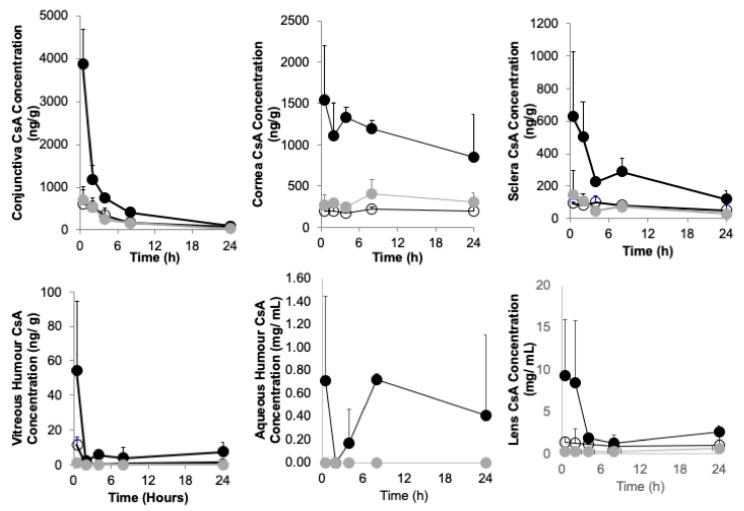
Ocular pharmacokinetics after the topical ocular dosing of NM133 and Restasis formulations to a healthy rabbit model (mean ± s.d., *n* = 3 per group); animals were dosed with a single dose of 25 µL of NM133 0.05%A (●), 25 µL of NM133 0.01%A (●) or 25 µL of Restasis 0.05% emulsion (◯). NM133 0.05%A delivers significantly more CsA to the ocular tissues than Restasis (*p* < 0.05). There was no plasma exposure to CsA with the NM133 formulations.

NM133, an aqueous CsA eye drop formulation, enables a significantly higher level of cyclosporine A to penetrate the ocular surface tissues when compared to the Restasis emulsion formulation (Figure 2 and Table 8). Levels of cyclosporine A that inactivate T-cells in vitro range from 10 to 500 ng mL^−1^ [25], hence achieving tissue levels in excess of 500 ng g^−1^—as has been demonstrated with both NM133 0.05%A and NM133 0.01%A (Figure 2 and Table 8)—and should result in pharmacological efficacy in Dry Eye patients, if the data is replicated in humans. The rabbit is an excellent model for the human eye, when compared to rodent eyes, as it is comparable in size and anatomy. There was no plasma exposure to CsA from any of the NM133 formulations (limit of detection = 0.4 ng mL^−1^) and virtually all of the Restasis dosed animals also showed no evidence of plasma exposure. However, at the four-hour time point, one of the Restasis dosed animals out of three had a plasma CsA level of 39 ng mL^−1^. Topical ocular MET formulations are able to deliver high concentrations of CsA to the ocular tissues without evidence of plasma exposure.

### 3.3. Ocular Tolerability

All animals tolerated the MET (no discomfort up to a concentration of 2% *w*/*v* and one animal dosed at the concentration of 4% *w*/*v* experienced minimal to mild discomfort). In non-GLP preclinical studies the MET polymer was well tolerated up to a concentration 4% *w*/*v*; a concentration that is over four times the concentration of the MET polymer contained in NM133 0.05%. 

The blink rate is shown in Table 9, and there was no significant difference in the blink rate observed on instillation of the Restasis and NM133 formulations. It is noteworthy that, while animal numbers were too low to record changes in ocular tolerability (measured by changes in blink rate), as the incidence of ocular eye pain with Restasis is 27% [7]; the concentration of active also had no significant effect on the blink rate. This suggests that the eye pain felt by Ikervis and Restasis patients may indeed stem from the vehicle as stated above.

## 4. Discussion

It has been demonstrated that the MET polymer may be used to prepare CsA eye drops comprised of MET nanoparticles encapsulating CsA (Figure 1, Table 2). The formulations dispersed in PBS were not selected for further evaluation as charge screening led to larger particle sizes and polydisperse systems (Table 2), due essentially to charge screening by the high salt levels in the formulation. Formulations prepared in the non-ionic tonicity agent glycerol had a small particle size (<100 nm) and were not polydisperse (PDI~0.4), due to an absence of charge screening. High pressure homogenization reduced particle size when carried out at 15 cycles, with particle size increasing after high pressure homogenization was carried out above 15 cycles (Figure S1 in Supplementary Information), and so future formulations will be prepared with 15 cycles or less of high pressure homogenization. The MET polymer increases CsA absorption in ocular tissues on topical ocular administration (Figure 2). This correlates well with our findings that the MET polymer significantly increases the absorption of tacrolimus on topical ocular administration [21] and is able to deliver high levels of prednisolone into the aqueous humour from a comparatively low dose [4]. It is clear that the MET polymer is a penetration enhancer and other studies have shown the drug to be available and pharmacologically active when delivered using the MET polymer [4].The possible patient benefit could arise in patients requiring higher tissue levels of CsA in order to achieve a pharmacological response [12], as detailed above, and these patients will benefit from the increased drug absorption experienced with NM133. Secondly, patient benefit could arise from a lower dosing frequency (28 μL of a 0.05% NM133 once daily as opposed to 28 μL of Restasis 0.05% dosed twice daily) that still achieves adequate drug levels in the tissues (Figure 2); a reduced dosing frequency will also limit the actual number of eye pain events. The MET vehicle used in NM133 is well tolerated in rabbits up to a concentration of 4% *w*/*v*.

**Figure 3:**
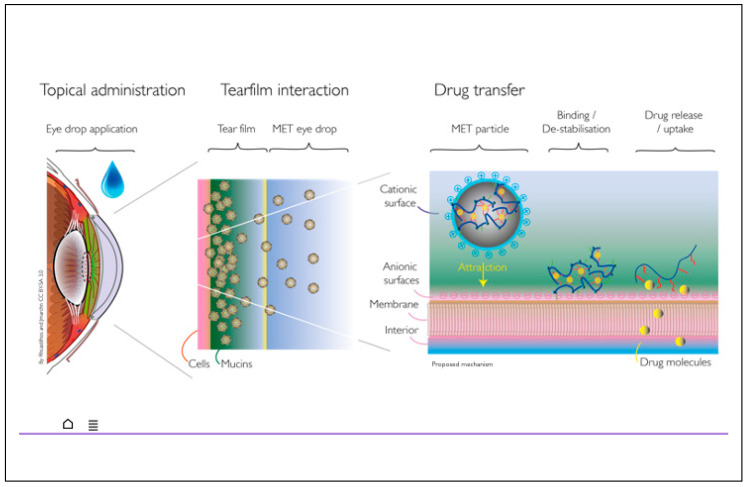
Hypothesised mechanism of action involving mucoadhesion and destabilization of drug carrying nanoparticles at the ocular surface.

The hypothesized reason for this greater penetration with NM133, when compared to the currently marketed oil in water emulsions, is the partitioning of CsA out of the oil phase (within which it is very soluble) and into the aqueous disperse phase is a comparatively high energy process, when compared to the partitioning of CsA from the MET nanoparticles into the disperse phase; the latter would happen as the MET nanoparticles are mucoadhesive [17] and, thus, the MET nanoparticles would be destabilized on adherence to the ocular mucosal surface, releasing drug into the disperse phase in close proximity to the ocular epithelium (Figure 3). Once in the disperse phase, the CsA would then partition into the lipid layers of the ocular tissues.

It is not inconceivable to argue that the presence of an oil phase within ocular emulsion formulations would effectively limit the penetration of CsA into the ocular tissues, as drug residence within the oil phase is energetically favorable. 

The MET excipient in NM133 is superior to a variety of delivery systems (emulsions [26], non-aqueous vehicles [27] and other polymeric micelles [28]), with respect to delivery of CsA to surface ocular tissues (Table 10), with unprecedented penetration enhancement coupled with a lack of ocular irritation. A useful method to compare the penetration enhancement afforded by the formulation excipients is to compare the Cmax per µg dosed (Table 10). NM133 is 2–3 times more efficient at delivering CsA to the cornea when compared to NOVA2007, 4–10 times more efficient at delivering CsA to the cornea when compared to Restasis, at least 4 times more efficient at delivering CsA to the cornea when compared to Cequa (comparing data obtained with NM133 at the two hour time point with data obtained using Cequa at the one hour time point), 9–12 times more efficient at delivering CsA to the cornea when compared to Cyclasol and 20–26 times more efficient at delivering CsA to the cornea when compared to the formulation published by Novaliq.

When considering the delivery to the conjunctiva using the same metric, NM133 is 7–11 times more efficient at delivering CsA to the conjunctiva when compared to NOVA22007, 7–9 times more efficient at delivering CsA to the conjunctiva when compared to Restasis and over 2 times more efficient at delivering CsA to the conjunctiva when compared to Cequa (comparing data obtained with NM133 at the two hour time point with data obtained using Cequa at the one hour time point). Levels of cyclosporine A that inactivate T-cells in vitro range from 10 to 500 ng mL^−1^ [25], hence achieving tissue levels in excess of 500 ng g^−1^—as has been demonstrated with NM133 when dosed at the 0.05% *w*/*v* or 0.01% *w*/*v* strengths (Figure 2)—and should result in efficacy in Dry Eye patients at a lower daily dose than would be achieved with Restasis eye drops. This lower daily dose could theoretically lead to a reduction in local adverse events. While all the vehicles used by others enable the hydrophobic CsA to be incorporated into aqueous media, they do not behave as penetration enhancers to the same extent as the MET polymer.

Furthermore, NM133 does not contain the surfactants and oils that result in the ocular discomfort felt by patients receiving the Restasis or Ikervis emulsion formulations and will not cause the loss of visual acuity [11] which is normally experienced when patients instil opaque emulsion formulations topically to the eye. In non-GLP preclinical studies, Nanomerics’ MET was well tolerated at the concentration above 4% *w*/*v*, a concentration that is over four times the concentration of Nanomerics’ MET contained in NM133. 

## 5. Conclusions

A new CsA eye drop formulation has been developed with an ocular mucoadhesive penetration enhancer—the MET polymer—which significantly increases drug levels by at least 5–6-fold in the cornea and conjunctiva respectively, when compared to commercial formulations. The penetration enhancer forms optically clear aqueous eye drops and thus may become useful in the formulation of hydrophobic drugs into topical ocular formulations. Furthermore, the MET excipient is non-irritant at the relevant concentrations.

The demonstration that the MET polymer not only enables the aqueous incorporation of hydrophobic drugs to levels compatible with clinical use but is also a significant penetration enhancer will allow the MET to be used in other ophthalmic applications, and these applications are currently being pursued.

## Figures and Tables

**Table 1:** NM133 Formulations Prepared.

Formulation Name	MET (% *w*/*v*)	Cyclosporine A (% *w*/*v*)	Diluent	Method of Preparation
NM133 0.01% A *	0.75	0.01	Glycerol (3.1% *w*/*v*), pH = 7.4	Method I
NM133 0.05% A	0.75	0.05	Glycerol (3.1%*w*/*v*), pH = 7.4	Method I
NM133 0.01% B	0.75	0.01	Glycerol (2.7% *w*/*v*), pH = 7.0	Method II
NM133 0.02% A	0.75	0.02	Glycerol (2.7% *w*/*v*), pH = 7.0	Method II
NM133 0.05% B	0.75	0.05	Glycerol (2.7% *w*/*v*), pH = 7.0	Method II
NM133 0.08% A	0.75	0.08	Glycerol (1.0% *w*/*v*)	Method IV
NM133 0.08% B	0.75	0.08	Glycerol (1.0% *w*/*v*)	Method IV
NM133 0.1% A	0.75	0.1	Glycerol (1.0% *w*/*v*)	Method IV
NM0133 0.05% C	0.375	0.05	Phosphate buffered saline (PBS, pH = 7.4)	Method III
NM0133 0.08% C	0.60	0.08	PBS (pH = 7.4)	Method III
NM0133 0.08% D	0.75	0.08	PBS (pH = 7.4)	Method III
NM0133 0.1% B	0.75	0.1	PBS (pH = 7.4)	Method III

* Letters A–D are simply used to distinguish the different methods of preparation used for formulations of similar strength.

**Table 2:** NM133 Formulation Characteristics, mean ± s.d. from three independent samples.

Formulation Strength	Nominal MET Concentration (mg mL^−1^)	CsA Conc. (mg mL^−1^)	z-Average Mean Particle Size (nm)/PDI	Zeta Potential (mV)	Viscosity mPa.s	pH	Osmolarity (mOsm L^−1^)	Disperse Phase
0.01% B	7.5	0.094 ± 0.001	39 ± 1.6/ 0.435 ± 0.004	+34 ± 0.7	1.338 ± 0.040	6.90 ± 0.0	313 ± 9	2.7% glycerol in water, pH adjusted to pH = 7
0.02% A	7.5	0.201 ± 0.007	37 + 0.7/ 0.414 ± 0.022	+26 ± 1.1	1.230 ± 0.040	7.0 ± 0.0	320 ± 3	2.7% glycerol in water, pH adjusted to pH = 7
0.05% B	7.5	0.510 ± 0.0004	36 ± 1.2/ 0.395 ± 0.020	+33 ± 1.9	1.303 ± 0.052	6.9 ± 0.0	323 ± 9	2.7% glycerol in water, pH adjusted to pH = 7
0.05% C	3.75	0.497 ± 0.0062	835 ± 43/ polydisperse (PDI > 0.6)	ND	ND	6.8 ± 0.3	311 ± 1	PBS, pH = 7
0.08% C	7.5	0.782 ± 0.0088	255 ± 96/ polydisperse (PDI > 0.6)	ND	ND	ND		PBS, pH = 7
0.1% B	7.5	1.02 ± 0.0098	806 ± 245/ polydisperse (PDI > 0.6)	ND	ND	7.0 ± 0.2	304 ± 1	PBS, pH = 7

**Table 3:** NM133 Storage at Elevated Temperature (40 °C), mean ± s.d. from three independent samples.

Formulation Strength	Day 0						Day 28					
	CsA Conc. (mg mL^−1^)	pH	Osmolarity (mOsm L^−1^)	Viscosity (mPa.s)	Zeta Potential (mV)	z-Average Mean Particle Size (nm)/PDI	CsA Conc. (mg mL^−1^)	pH	Osmolarity (mOsm L^−1^)	Viscosity (mPa.s)	Zeta Potential (mV)	z-Average Mean Size (nm)/PDI
0.01% B	0.094 ± 0.001	6.93 ± 0.06	313 ± 9	1.338 ± 0.040	+34 ± 0.7	39 ± 1.6/ 0.435 ± 0.004	0.106 ± 0.008	ND	328 ± 8 *	1.210 ± 0.006 *	+20 ± 1.4 *	28 ± 1.7/ 0.408 ± 0.007
0.02% A	0.201 ± 0.007	6.93 ± 0.06	320 ± 3	1.23 ± 0.004	+26 ± 1.1	37 + 0.7/ 0.414 ± 0.022	0.228 ± 0.018 *	ND	383 ± 9 *	1.207 ± 0.005	+20 ± 1.5 *	28 ± 1.0/ 0.411 ± 0.0134
0.05% B	0.510 ± 0.004	6.93 ± 0.06	323 ± 9	1.303 ± 0.052	+33 ± 1.9	36 ± 1.2/ 0.395 ± 0.020	0.533 ± 0.031	ND	349 ± 7 *	1.339 ± 0.020	+41 ± 3.2 *	24 ± 0.6/ 0.398 ± 0.005

* significantly different (*p* < 0.05) than the freshly prepared formulation.

**Table 4:** NM133 Storage at Room Temperature (18–23 °C), mean ± s.d. from three independent samples.

Formulation Strength	Day 0						Day 28					
	CsA Conc. (mg mL^−1^)	pH	Osmolarity (mOsm L^−1^)	Viscosity (mPa.s)	Zeta Potential (mV)	z-Average Mean Particle Size (nm)/PDI	CsA Conc. (mg mL^−1^)	pH	Osmolarity (mOsm L^−1^)	Viscosity (mPa.s)	Zeta Potential (mV)	z-Average Mean Particle Size (nm)/PDI
0.01% B	0.094 ± 0.001	6.9 ± 0.0	313 ± 9	1.338 ± 0.040	+34 ± 0.7	39 ± 1.6/ 0.435 ± 0.004	0.100 ± 0.003 *	ND	324 ± 11	1.215 ± 0.003 *	+23 ± 7	32 ± 2.6/ 0.380 ± 0.038
0.02% A	0.201 ± 0.007	7.0 ± 0.0	320 ± 3	1.230 ± 0.004	+26 ± 1.1	37 + 0.7/ 0.414 ± 0.022	0.203 ± 0.008	ND	339 ± 4 *	1.208 ± 0.002	+26 ± 2.6	29 ± 0.5/ 0.400 ± 0.016
0.05% B	0.510 ± 0.004	6.9 ± 0.0	323 ± 9	1.303 ± 0.052	+33 ± 1.9	36 ± 1.2/ 0.395 ± 0.020	0.506 ± 0.022	ND	330 ± 1	1.358 ± 0.005 *	+31 ± 3.8	28 ± 0.5/ 0.317 ± 0.018

* significantly different (*p* < 0.05) than the freshly prepared formulation.

**Table 5:** NM133 Storage at Refrigeration Temperature (5–6 °C), mean ± s.d. from three independent samples.

Formulation Strength	Day 0						Day 28					
	CsA Conc. (mg mL^−1^)	pH	Osmolarity (mOsm L^−1^)	Viscosity (mPa.s)	Zeta Potential (mV)	z-Average Mean Particle Size (nm)/PDI	CsA Conc. (mg mL^−1^)	pH	Osmolarity (mOsm L^−1^)	Viscosity (mPa.s)	Zeta Potential (mV)	z-Average Mean Particle Size (nm)/PDI
0.01% B	0.094 ± 0.001	6.9 ± 0.0	313 ± 9	1.338 ± 0.040	+34 ± 0.7	39 ± 1.6/ 0.435 ± 0.004	0.097 ± 0.005	ND	319 ± 13	1.234 ± 0.032 *	+35 ± 0.1	33 ± 1.9/0.387 ± 0.062
0.02% A	0.201 ± 0.007	7.0 ± 0.0	320 ± 3	1.230 ± 0.004	+26 ± 1.1	37 + 0.7/ 0.414 ± 0.022	0.202 ± 0.011	ND	331 ± 1 *	1.234 ± 0.007	+37 ± 0.9 *	33 ± 0.4/0.409 ± 0.004
0.05% B	0.510 ± 0.004	6.9 ± 0.0	323 ± 9	1.303 ± 0.052	+33 ± 1.9	36 ± 1.2/ 0.395 ± 0.020	0.502 ± 0.024	ND	324 ± 5	1.209 ± 0.015*	+36 ± 3.2	30 ± 0.3/0.385 ± 0.041

* significantly different (*p* < 0.05) than the freshly prepared formulation.

**Table 6:** NM133 0.08% C Storage Stability Studies Following Freeze Thaw Cycling, mean ± s.d. from three independent samples.

Storage Days	Cyclosporine A Concentration (Mean ± s.d., mg mL^−1^)	Z-Average Mean Particle Size (nm) *	Storage Temperature
0	0.782 ± 0.088	255 ± 96	Not Applicable
1–2	0.725 ± 0.026	336 ± 136	−20 °C
3–4	0.907 ± 0.108	332 ± 97	4 °C
5–6	0.724 ± 0.070	386 ± 96	40 °C
7–8	0.863 ± 0.113	381 ± 134	−20 °C
9–10	0.820 ± 0.068	325 ± 10	4 °C
11–12	0.773 ± 0.058	294 ± 37	40 °C
13–14	0.787 ± 0.107	332 ± 65	−20 °C
15–16	0.697 ± 0.164	279 ± 73	4 °C
17–18	0.834 ± 0.067	374 ± 118	40 °C

* All formulations were polydisperse with a polydispersity index exceeding or equal to 0.6.

**Table 7:** Results of British Pharmacopoeia Sterility Testing on Sterile Filtered NM133 0.05%A and the NM133 Vehicle.

Formulation	Number of Colony Forming Units		
	Freshly Prepared	24 h after Sterile Filtration	5 Days after Sterile Filtration
NM133 0.05%A Sample 1	1	0	1
NM133 0.05%A Sample 2	0	0	0
NM133 0.05%A Sample 3	3	0	0
MET 0.75% Sample 1	0	0	0
MET 0.75% Sample 2	0	0	0

**Table 8:** AUC_0–24_ in selected tissues following the topical ocular dosing of NM133 and Restasis formulations *.

Formulation	Cyclosporine A Topical Ocular Dose (μg)	AUC_(0–24)_ (ng.h/g)			Cmax (ng mL^−1^)		
		Cornea	Conjunctiva	Sclera	Cornea	Conjunctiva	Sclera
NM133 0.05% A	12.5	25,780	12,046	5879	1546	3864	627
NM133 0.01% A	2.5	8024	3988	1372	410	703	146
Restasis 0.05%	12.5	4726	4813	1729	216	608	101

* Comparatively very low levels of drug were seen in the aqueous humor, vitreous humour and lens with all formulations, as shown in Figure 2.

**Table 9:** Blink Rate Immediately Following the Topical Ocular Administration of Cyclosporine A Formulations.

Formulation	Blink Rate per Minute
NM0133 0.05% A	5.3 ± 2.76
NM0133 0.01% A	4.7 ± 2.25
Restasis	6.1 ± 2.56

**Table 10:** Comparative drug deposition levels in the conjunctiva and cornea following the administration of a single topical ocular dose of various cyclosporine formulations.

Formulation	Total Ocular Dose (μg)	Cornea Cmax (ng g^−1^)	Conjunctiva Cmax (ng g^−1^) *	Dose Strength (% *w*/*v*)	Dose Volume (μL)	Calculated Corneal Cmax per μg Dosed	Calculated Conjunctival Cmax per μg Dosed	Reference
NOVA22007	50	2691	1914	0.1	50	54	38	[26]
NOVA22007	25	1372	696	0.05	50	55	29	[26]
Comparative Restasis Data from the Cornea 2013 Study	25	748	848	0.05	50	30	34	[26]
Cequa * (one hour time point)	35	828	1417	0.1	35	24	40	[28]
NM133	12.5	1546	3864	0.05	25	124	309	Current study
NM133	2.5	409	703	0.01	25	164	281	Current study
Comparative Restasis Data in Nanomerics’ Studies	12.5	216	608	0.05	25	17	49	Current study
Cyclasol	100	1326	Not given	Not given	Not given	13	N/A	[27]
Oil In water emulsion tested by Novaliq	100	633	Not given	Not given	Not given	6	N/A	[27]

* data taken from the one hour time point after dosing. Comparative NM133 levels at the two hour time point were 1182 ng/g for the conjunctiva with about a third of the topical ocular dose given (12.5 vs. 35 μg) and 1109 ng/g for the cornea with about a third of the topical ocular dose given (12.5 vs. 35 μg).

## Data Availability

The raw data presented in this study are available on request from the corresponding author. The data are not publicly available due to privacy restrictions.

## Supplementary Materials

The following are available online at https://www.mdpi.com/article/10.3390/pharmaceutics13050744/s1, Figure S1: HPLC Chromatogram of CsA (1.25 mg mL^−1^). Figure S2: Particle size distribution following the application of increasing high pressure homogenization cycles B0 (before high pressure homogenization)—B35 (after 35 cycles). Above 15 cycles a second larger peak appears. High pressure homogenization was thus limited to 15 cycles in Method II. Table S1: Preparation of CSA working standard solutions. Table S2: Preparation CSA calibration standards. Table S3: Ion channel detector setting for the LC-MS/MS analysis of CsA.
