# Supplementary material for: Polymeric Micelles for the Enhanced Deposition of Hydrophobic Drugs into Ocular Tissues, without Plasma Exposure
Source: Pharmaceutics. 2021 May 18;13(5):744. doi: 10.3390/pharmaceutics13050744 (PMC8157576; doi:10.3390/pharmaceutics13050744)
Supplement: Supplementary file 1 [file pharmaceutics-13-00744-s001.zip › pharmaceutics-1200675-supplementary.pdf]

pharmaceutics

MDPI

Supplementary Information: Polymeric micelles for the enhanced deposition of hydrophobic drugs into ocular tissues, without plasma exposure

Ijeoma F. Uchegbu, Jan Breznikar, Alessandra Zaffalon, Uche Odunze and Andreas G. Schatzlein

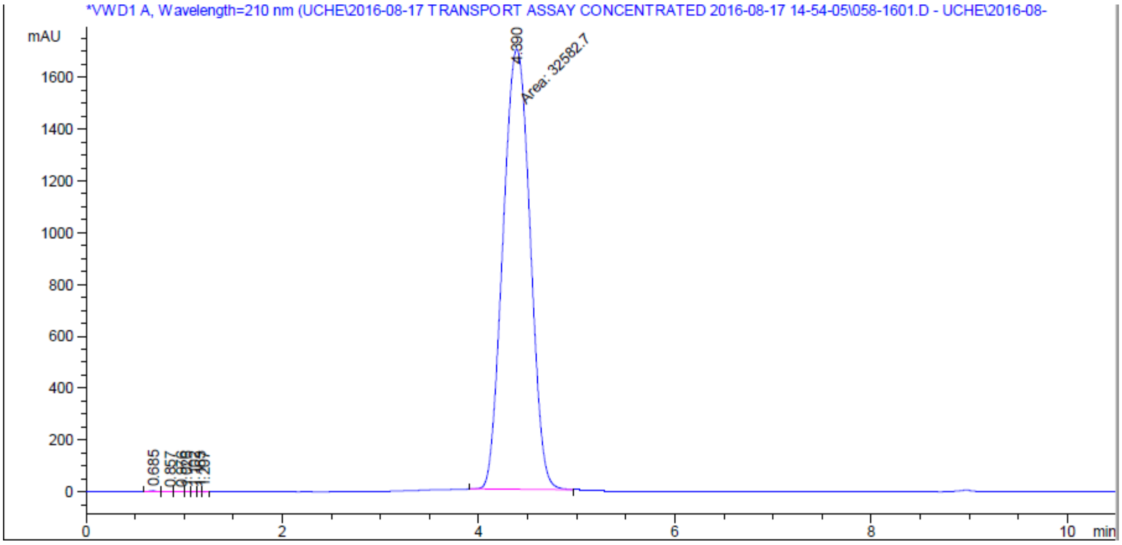

Figure 1. HPLC Chromatogram of CsA (1.25 mg mL^{-1}).

Table S1. Preparation of CSA working standard solutions.

**Code**	**Dilutions**			**Final concentration**
	Take amount (µL)	From	Add methanol (µL)	CSA (ng/mL)
**WS14**	200	**CsA STD**	0	1,000,000
**WS13**	100	**WS14**	100	500000
**WS12**	80	**WS13**	120	200000
**WS11**	100	**WS12**	100	100000
**WS10**	100	**WS11**	100	50000
**WS9**	100	**WS10**	100	25000
**WS8**	100	**WS9**	100	12500
**WS7**	100	**WS8**	100	6250
**WS6**	100	**WS7**	100	3125
**WS5**	100	**WS6**	100	1562.50
**WS4**	100	**WS5**	100	781.25
**WS3**	100	**WS4**	100	390.63
**WS2**	100	**WS3**	100	195.31
**WS1**	100	**WS2**	100	97.67

**Code**	**Dilutions**			**Final concentration**
	Take amount (µL)	From	Add methanol (µL)	CSA (ng/mL)
**WS0**	100	**WS1**	100	48.83

Table S2. Preparation CSA calibration standards.

**Sample Number**	**Spike volume (µL)**	**From**	**Final concentration (ng/mL)**
**Std S14**	1	**WS14**	10000
**Std S13**	1	**WS13**	5000
**Std S12**	1	**WS12**	2000
**Std S11**	1	**WS11**	1000
**Std S10**	1	**WS10**	500
**Std S9**	1	**WS9**	250
**Std S8**	1	**WS8**	125
**Std S7**	1	**WS7**	62.5
**Std S6**	1	**WS6**	31.25
**Std S5**	1	**WS5**	15.63
**Std S4**	1	**WS4**	7.81
**Std S3**	1	**WS3**	3.91
**Std S2**	1	**WS2**	1.95
**Std S1**	1	**WS1**	0.97
**Std S0**	1	**WS0**	0.49

Table S3. Ion channel detector setting for the LC-MS/MS analysis of CsA.

**Ion Channels**	**Precursor Ion → Product Ion**	**MS1/MS2 resolution**	**Dwell**	**Fragmentator (V)**	**Collision Exit Potential (V)**	**Cell Acceleration Voltage (V)**
**CSA**	1224.9 → 1112.7	widest/widest	370	350	70	1
**CSA-d12**	1236.9 → 1124.2	widest/widest	250	350	75	1

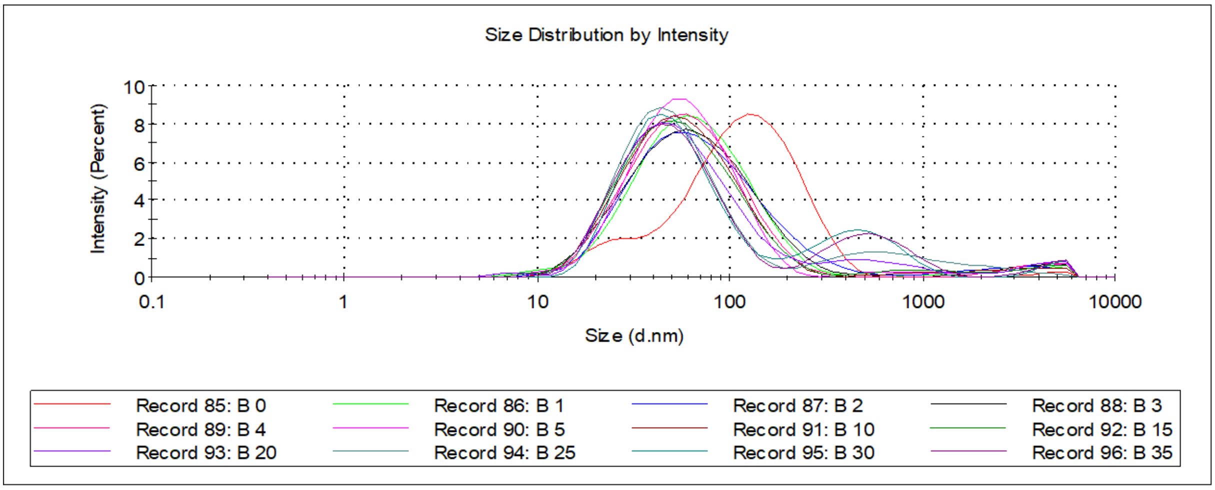

Figure S2. Particle size distribution following the application of increasing high pressure homogenisation cycles B0 (before high pressure homogenisation) – B35 (after 35 cycles). Above 15 cycles a second larger peak appears. High pressure homogenisation was thus limited to 15 cycles in Method II.
